# Case report: Adult-onset neuronal intranuclear inclusion disease with an amyotrophic lateral sclerosis phenotype

**DOI:** 10.3389/fnins.2022.960680

**Published:** 2022-08-10

**Authors:** Masako Fujita, Tatsuya Ueno, Yasuo Miki, Akira Arai, Hidekachi Kurotaki, Koichi Wakabayashi, Masahiko Tomiyama

**Affiliations:** ^1^Department of Neurology, Aomori Prefectural Central Hospital, Aomori, Japan; ^2^Department of Neuropathology, Hirosaki University Graduate School of Medicine, Hirosaki, Japan; ^3^Department of Pathology, Aomori Prefectural Central Hospital, Aomori, Japan; ^4^Department of Neurology, Hirosaki University Graduate School of Medicine, Hirosaki, Japan

**Keywords:** neuronal intranuclear inclusion disease, amyotrophic lateral sclerosis, sporadic, autopsy, muscle atrophy

## Abstract

Amyotrophic lateral sclerosis (ALS) is one of the differential diagnoses of diseases that occur in adulthood and lead to progressive generalized muscle weakness. Neuronal intranuclear inclusion disease (NIID) is a disease in which histopathologically eosinophilic nuclear inclusion bodies are found in various systems. Both familial and sporadic forms of the disease have been reported. Most cases of sporadic NIID are of the dementia type, in which the main symptom is dementia at the first onset. Familial NIID is more diverse, with the main dominant symptoms being muscle weakness (NIID-M), dementia (NIID-D), and parkinsonism (NIID-P). Furthermore, recently, a GGC-repeat expansion in the Notch 2 N-terminal like C (*NOTCH2NLC*) gene, which produces a toxic polyglycine-containing protein (uN2CpolyG) in patients with NIID, has been associated with the pathogenesis of ALS. These results suggest that sporadic NIIDs may have more diverse forms. To date, no autopsy cases of NIID patients with an ALS phenotype have been reported. Here, we describe the first autopsy case report of a patient with sporadic NIID who had been clinically diagnosed with ALS. A 65-year-old Japanese man with no family history of neuromuscular disease developed progressive muscle atrophy and weakness in all limbs. The patient was diagnosed with ALS (El Escoriral diagnostic criteria: probable ALS, laboratory-supported ALS). He had no cognitive dysfunction or neuropathies suggestive of NIID. He required respiratory assistance 48 months after onset. He died of pneumonia at the age of 79 years. Postmortem examinations revealed neuronal loss in the spinal anterior horns and motor cortex. In these affected regions, eosinophilic, round neuronal intranuclear inclusions were evident, which were immunopositive for ubiquitin, p62, and uN2CpolyG. No Bunina bodies or TDP-43-positive inclusions were observed in the brain or spinal cord. Our findings suggest that a small proportion of patients with NIID can manifest a clinical phenotype of ALS. Although skin biopsy is commonly used for the clinical diagnosis of NIID, it may also be useful to identify cases of NIID masquerading as ALS.

## Introduction

There are a variety of neuromuscular diseases that cause progressive generalized muscle weakness; of these, amyotrophic lateral sclerosis (ALS) is a disease that presents with both upper and lower motor neuron signs. Clinically, the degree of disturbance of upper and lower motor neurons in ALS varies between cases. The characteristic pathology of sporadic ALS is the loss of upper and lower motor neurons and the presence of Bunina bodies and TAR DNA binding protein 43 (TDP-43)-positive skein-like or round inclusions in lower motor neurons (Yoshida, 2019). In addition, approximately half of ALS patients have demonstrable cognitive deficits of varying degrees. Moreover, 15–20% of patients have severe cognitive dysfunction to be clinically diagnosed as dementia (Lomen-Hoerth et al., 2003; Murphy et al., 2007; Witgert et al., 2010). The pattern of cognitive dysfunction is similar to that of frontotemporal lobar degeneration (FTLD): behavioral changes, decreased motivation, and language problems. ALS and in some FTLD cases share a common molecular pathogenesis; in that inclusions containing the same constituent proteins (TDP-43, fused in sarcoma (FUS)) as the inclusions that appear in motor neurons in ALS are found as well as in cortical neurons in FTLD (Mackenzie et al., 2010).

Neuronal intranuclear inclusion disease (NIID) is a neurodegenerative disease with slow progression and very low incidence. In this disorder, cells of the central nervous system (CNS), peripheral nervous system, and autonomic nervous system, as well those of the visceral organs (Lindenberg et al., 1968; Sone et al., 2016) present characteristic eosinophilic hyaline intranuclear inclusions. Sporadic and familial forms of the disease have been previously described. In the sporadic form, dementia is the most prominent initial symptom in the designated dementia-dominant group (Sone et al., 2016). Several clinical presentations of NIID have been described that include, either alone or combined symptoms such as cerebellar ataxia (Kimber et al., 1998; Zannolli et al., 2002; Kotani et al., 2021), parkinsonism (Liu et al., 2008), peripheral neuropathy (Sone et al., 2005), autonomic dysfunction (Sone et al., 2005), stroke-like episodes (Fujita et al., 2017; Lin et al., 2020; Huang et al., 2021) and encephalitic episodes (Liu et al., 2008; Li et al., 2020; Huang et al., 2021). This variety in presenting symptoms can hinder clinicians' ability to make an antemortem diagnosis.

A high-intensity signal at the corticomedullary junction on diffusion-weighted imaging (DWI) and skin biopsy showing intranuclear inclusions in dermal cells can be indicative of NIID (Sone et al., 2011, 2014). In recent years, a GGC-repeat expansion in the *NOTCH2NLC* gene has been identified as a pathogenic cause of NIID (Sone et al., 2019). Moreover, several research groups have reported the association of expanded GGC repeats in *NOTCH2NLC* with neurodegenerative disorders such as sporadic and familial essential tremor (Chen et al., 2020; Ng et al., 2020; Sun et al., 2020), sporadic and familial oculopharyngeal distal myopathy (Ogasawara et al., 2020; Yu et al., 2021), multiple system atrophy (Fang et al., 2020), Parkinson's disease (Ma et al., 2020; Shi et al., 2021), and ALS (Tian et al., 2019; Yuan et al., 2020). In NIID cases with cognitive dysfunction, the lower frontal assessment battery scores were more apparent than the mini-mental state examination. Some patients with NIID may also exhibit behavioral symptoms (Sone et al., 2016). Therefore, NIID and FTLD may have a similar presentation. Additionally, Bin et al. reported that *NOTCH2NLC* gene repeats were elongated in clinically diagnosed FTLD patients (Jiao et al., 2020). These results suggest the possibility that sporadic NIID has more diverse forms.

Thus, far, several cases of coexisting ALS and NIID pathology have been described (Kakita et al., 1997; Seilhean et al., 2004; Sugiyama et al., 2021); however, there have been no reports of only NIID pathology with an ALS phenotype. In this study, we report a patient who met the clinical criteria for ALS but had autopsy-confirmed NIID.

## Case description

A 65-year-old Japanese man presented with progressive weakness of the left upper and lower limbs for 22 months. He developed left thalamic hemorrhage spontaneously, followed by hemiplegia, sensory disturbance, and ataxia on the right side at the age of 64 years. His medical history included loss of the right thumb, middle finger, and ring finger owing to burns in his childhood; appendicitis; and lumbar spondylosis. Neuromuscular or neurodegenerative disorders were not observed among his family members.

On examination, the patient had muscle weakness in all extremities (Medical Research Council grades: 4 for right upper and lower limbs and 3–4 for proximal and distal portions of the left upper and lower limbs) and wasting of both arms, first dorsal interosseous muscles, thenar muscle, and legs. Fasciculations were observed in the tongue and both vastus medialis muscles. Additionally, paresthesia and ataxia of the right upper and lower limbs persisted after thalamic hemorrhage. Deep tendon reflexes were hypoactive and both plantar reflexes were extensors. No sensory impairment was observed in any extremity.

Needle electromyography showed active denervation (fibrillation potentials and positive sharp waves) and chronic neurogenic changes (long-duration and high-amplitude motor unit potentials [MUPs]; a reduced interference pattern with rapid firing; and reduced number of MUPs) in his biceps, first dorsal interosseous, vastus lateralis, and tibialis anterior muscles. Motor nerve conduction studies revealed compound muscle action potential reduction in the bilateral median nerves. Sensory nerve conduction study findings were normal (Table 1).

**Table 1:** Results of nerve conduction studies.

**Nerve**		**DL (ms)**	**CMAP (mV)**	**MCV (m/s)**	**F (ms)**	**SNAP (μV)**	**SCV (m/s)**
Median	R	4.1	2.5	48	29	15	51.3
	L	4.2	1.8	56.6	NE	19	55.2
Ulnar	R	2.5	3.6	47.1	27.8	11	53.3
	L	3.1	2.7	55.1	24.2	10	56.4
Tibial	R	4.4	2.7	45.5	55.8	-	-
	L	4.2	4	44.4	50.9	-	-
Sural	R					7.4	44.3
	L					6.9	49.2

*R, right side; L, left side; DL, distal latency; CMAP, compound muscle action potential; MCV, motor nerve conduction velocity; F, F-latency; SNAP, sensory nerve action potential; SCV, sensory nerve conduction velocity; NE, not evoked*.

Magnetic resonance imaging (MRI) of the cervical spine revealed mild cervical myelopathy. However, MRI with short T1 inversion recovery showed no intramedullary signal changes, which could not explain the progressive limb weakness.

**Figure 1:**
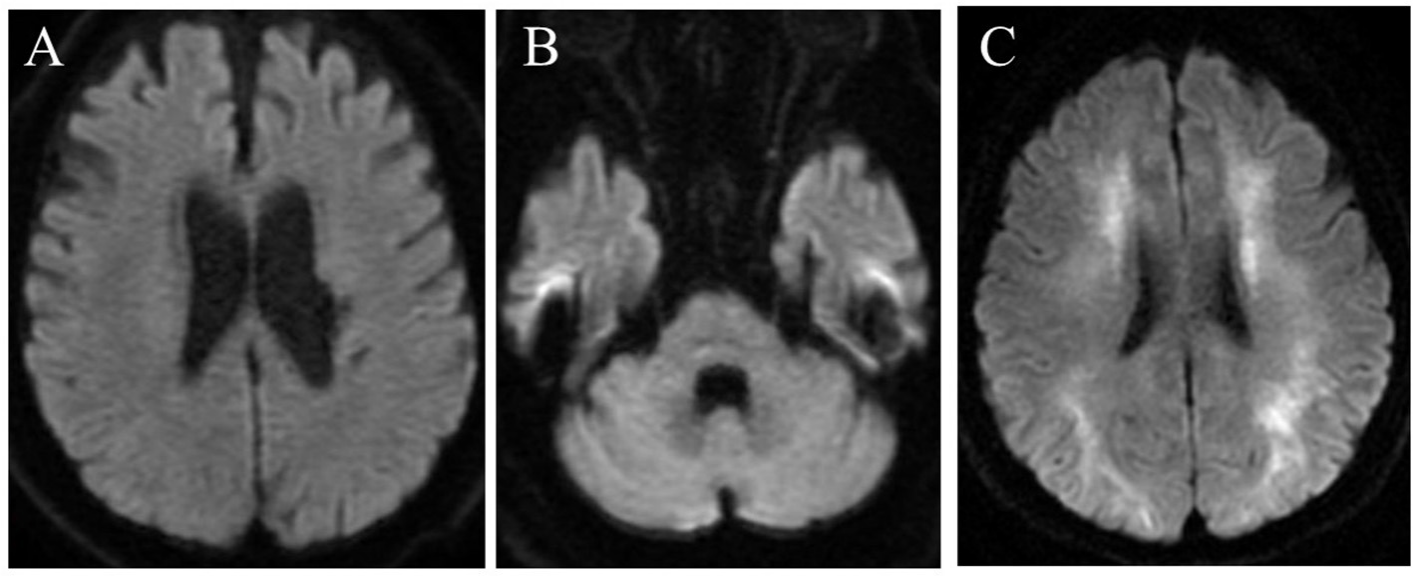
Brain diffusion-weighted magnetic resonance imaging in the present case (at the age of 76 years) **(A,B)** and in another NIID patient at our institution with typical imaging findings of corticomedullary junction **(C)**. No high-intensity signal is seen in the corticomedullary junctions **(A)** or cerebellar hemisphere **(B)**.

The patient met the El Escorial diagnostic criteria for probable ALS: laboratory-supported ALS with upper motor signs in one region and lower motor signs in three regions, as defined by electromyographic findings. Riluzole was initiated for ALS. The patient developed dysphagia and dyspnea owing to progressive weakness of the respiratory muscles and required artificial respiratory support and gastrostomy at the age of 68 years. Brain DWI at the age of 76 years showed no abnormalities in the cortical medullary junctions and cerebellar paravermis (Figures 1A,B) as seen in another NIID patient at our institution with typical imaging findings of corticomedullary junction (Figures 1A–C). There were no apparent episodes of cognitive impairment during the course of the disease. On the other hand, there were some atypical ALS signs, such as a relatively long disease course, including the fact that the patient was removed from the ventilator for some time during the day even after the mechanical ventilation initiation and the fact that upper motor neuron signs were not prominent. Neuropathy, muscular diseases, and spinal muscular atrophy were considered differential diagnoses, but nerve conduction test and electromyography showed negative results. Moreover, the patient had chronic intestinal pseudo-obstruction for many years. The patient died of pneumonia at the age of 79 years after a 15-year clinical course.

**Figure 2:**
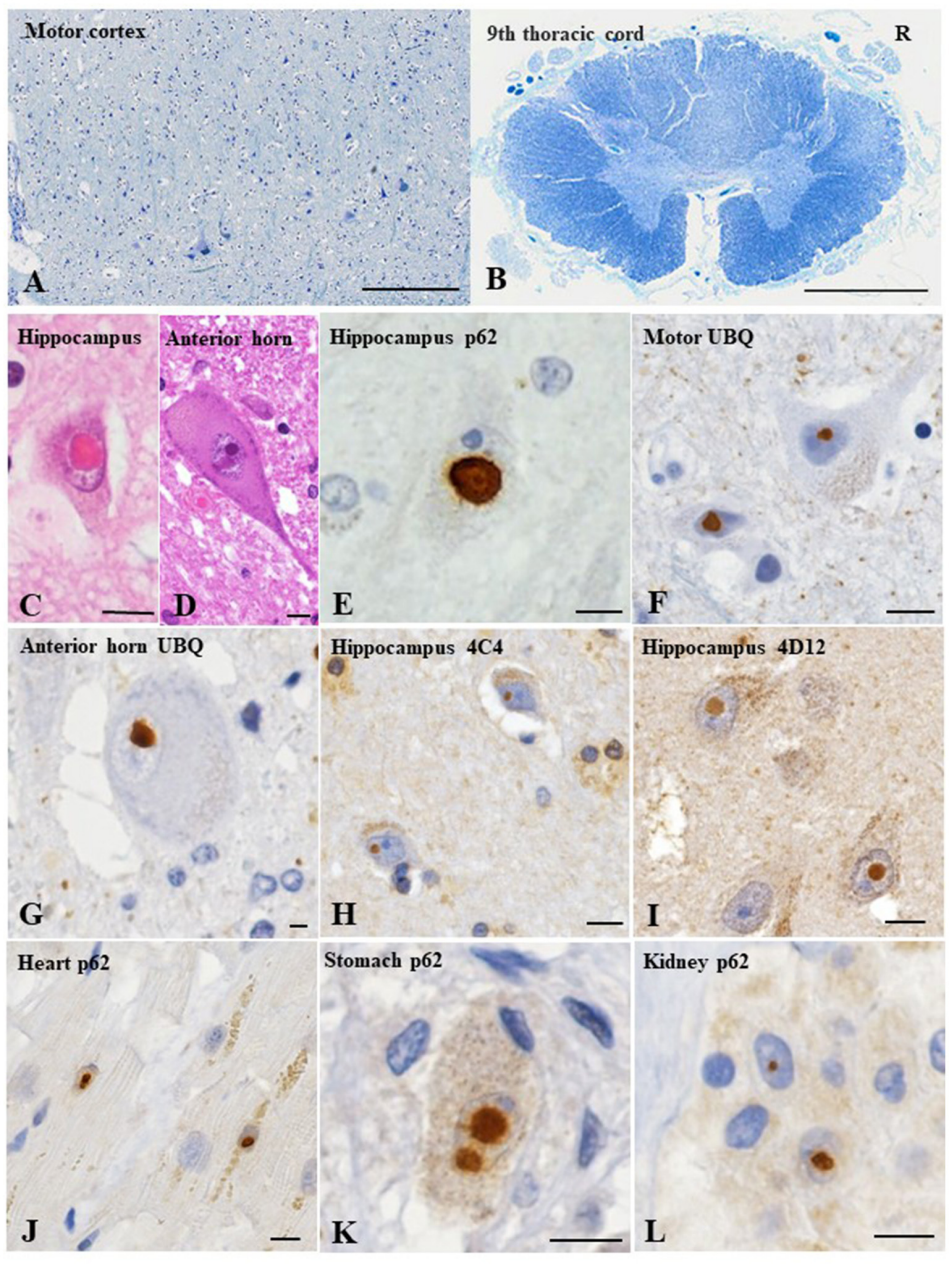
Pathological findings of the present case. The motor cortex shows slight loss of neurons **(A)**. Thoracic spinal cord shows myelin pallor of the lateral corticospinal tract bilaterally, more apparent on the right side (R), reflecting the left thalamic hemorrhage **(B)**. Hematoxylin and eosin staining reveals an eosinophilic, round, neuronal intranuclear inclusion in the hippocampus **(C)** and anterior horn of the lumbar cord **(D)**. Immunohistochemical evaluations reveal ubiquitin- and p62-immunopositive neuronal intranuclear inclusions in the hippocampus **(E)**, motor cortex **(F)** and spinal anterior horn **(G)**. In addition, uN2CpolyG immunoreactivity is confirmed by two specific antibodies (4C4 and 4D12) **(H, I)**. p62-positive neuronal intranuclear inclusions are evident in the cardiac muscle cells **(J)**, Auerbach's plexus of the stomach **(K)**, and renal tubule epithelial cells **(L)**. Klüver–Barrera staining **(A, B)**; p62 **(E, J–L)**; ubiquitin **(F, G)**; uN2CpolyG (4C4) **(H)**; uN2CpolyG (4D12) **(I)**. Bars = 100 μm **(A)**, 2 mm **(B)**, and 10 μm **(C–L)**.

**Figure 3:**
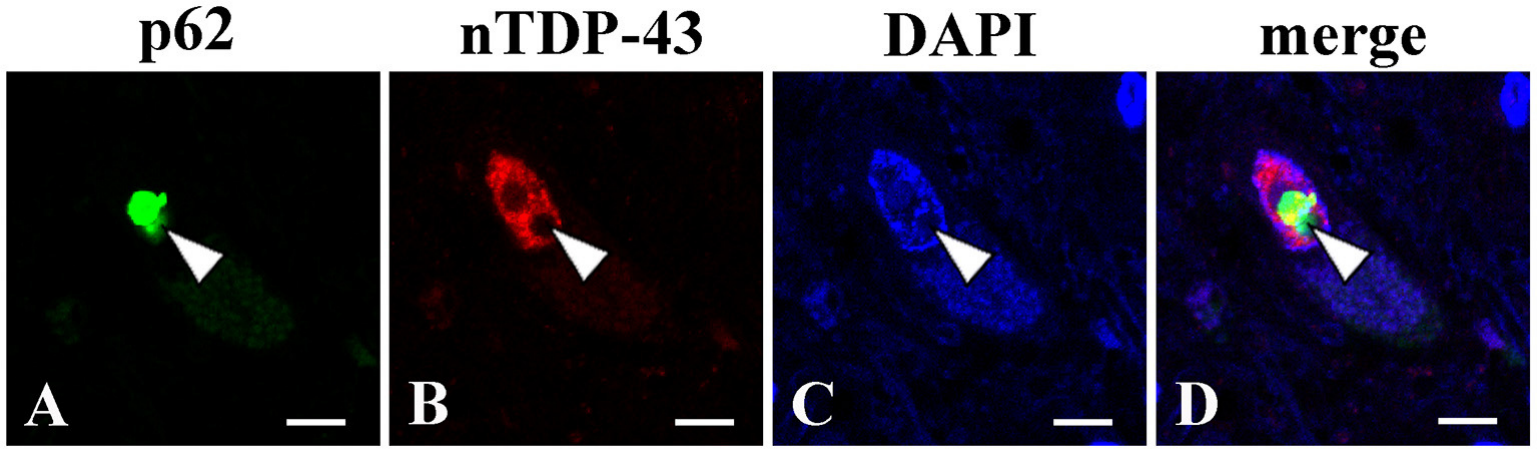
Double immunofluorescence analysis using the lumber spinal cord (L5). A p62 immunopositive-intranuclear inclusion **(A)** present with native TDP-43 (nTDP-43) **(B)** in the nucleus of anterior horn motor neuron. p62 **(A)**; nTDP-43 **(B)**; 4',6-diamidino-2-phenylindole (DAPI) **(C)**; merged image **(D)**. Bars = 10 μm.

At autopsy, the brain weighted 1,330 g before fixation. Macroscopic examinations showed no atrophy of the brain, while the spinal cord was atrophic with thinning and brownish discoloration of the anterior nerve roots. Histopathological evaluations revealed slight loss of neurons in the motor cortex (Figure 2A). Moderate to marked loss of neurons with gliosis was seen in the facial and hypoglossal nuclei and in the spinal anterior horn. No apparent neuronal loss was found in the oculomotor and Onuf's nuclei. The lateral corticospinal tracts degenerated bilaterally; however, the degeneration was more apparent on the right side (Figure 2B), reflecting left thalamic hemorrhage. This pyramidal tract degeneration was one of the pathologic substrates underlying altered bilateral extensor plantar responses. Hematoxylin and eosin staining showed the widespread occurrence of eosinophilic, round, neuronal intranuclear inclusions in the CNS. While these inclusions were particularly frequent in the hippocampus, they were also noted in the motor cortex, facial and hypoglossal nuclei, and anterior horn of the spinal cord, being immunopositive for ubiquitin, p62, and uN2CpolyG (4C4 and 4D12) (Figures 2C–I). However, inclusion bodies were not immunostained with antibodies against *FMR1* and polyglutamine. The results of other immunostaining reagents used to evaluate the immunoreactivity of nuclear inclusion bodies are listed in Supplementary material 1. In addition, we examined the visceral organs, confirming p62-positive inclusions in the cardiac muscle cells, Auerbach's plexus of the stomach, and epithelial cells in the renal tubules (Figures 2J–L). These findings allowed the diagnosis of NIID. There were no Bunina bodies or TDP-43 pathology including skein-like inclusions, round inclusions, and nuclear depletion of native TDP-43 in the brain and spinal cord. We further demonstrated that native TDP-43 was present in neuronal nuclei with p62-positive intranuclear inclusions, indicating that NIID pathology and TDP-43 pathology are independent (Figures 3A–D). Frozen tissue samples were not obtained in the present case.

## Discussion

The patient had a clinical course of progressive muscle weakness and met the El Escorial diagnostic criteria for ALS. However, autopsy confirmed the pathology of NIID but not that of ALS.

The clinical presentations of adult-onset NIID vary widely and can be divided into three main types based on dominant symptoms: dementia (NIID-D), limb weakness (NIID-M), and parkinsonism (NIID-P) (Sone et al., 2016; Tian et al., 2019). Most sporadic NIID cases are characterized by dementia being the first major clinical manifestation. Familial NIID cases are of several types. NIID-M is mainly familial, with the age of onset below 40 years. According to previous reports, patients with NIID-M present with diffuse limb muscle weakness with slow deterioration, distally dominant sensory disturbances, and autonomic dysfunction (Sone et al., 2016). In this subtype, dementia or leukoencephalopathy is mild and is not present in the first 20 years after the onset of NIID (Sone et al., 2016). Nerve conduction studies show slower conduction in both motor and sensory nerves, followed by compound muscle action potential and sensory nerve action potential reduction (Sone et al., 2016; Yuan et al., 2020). Histopathological findings show an increased presence of eosinophilic intranuclear inclusions in cells of the CNS and peripheral nervous system, as well as in somatic cells (Sone et al., 2016). The frequency of intranuclear inclusions in neurons is 5–30% and that in astrocytes is 10–30% (Sone et al., 2016). Although nuclear inclusions are widely distributed, they do not always correspond to neuronal loss, which varies from case to case and explains the diversity of the clinical syndrome. Boivin et al. indicated that uN2CpolyG (4C4) and anti-uN2C polyG (4D12) antibodies immunostained samples with neuronal interactions collected from NIID cases with an expansion of GGC repeats in *NOTCH2NLC* (Boivin et al., 2021).

In this case, the patient had diffuse limb muscle weakness without sensory disturbance or dementia, consistent with the diagnostic criteria for ALS. CNS findings were different from those for NIID and ALS. However, unlike typical NIID, most nuclear inclusions were distributed in neurons and only a few were distributed in glial cells, and there were no Bunina bodies or a TDP-43 pathology such as skein-like inclusions, round inclusions, and nuclear depletion of native TDP-43. Double immunolabeling demonstrated that TDP-43 pathology did not coexist with p62 pathology, indicating that NIID in this case is completely distinct from ALS. The patient presented with generalized muscle weakness, reflecting loss of motor neurons. The absence of ALS and presence of nuclear inclusions coinciding with neuronal loss suggest that the distribution of the nuclear inclusions caused symptoms consistent with the clinical presentation of ALS. Although we could not perform genetic testing, 4C4 and 4D12, which are immunopositive in the inclusion bodies of patients with expanded GGC repeats in *NOTCH2NLC*, were immunopositive in the present case. This finding suggests that our patient had this genotype. Importantly, in our case study, there was a lack of high-intensity signals in the corticomedullary junction on MRI-DWI, as reported in a high proportion of sporadic adult NIID patients. However, only 33% of familial NIID-M patients have this MRI-DWI change (Sone et al., 2016). Furthermore, a few sporadic cases with no brain abnormalities on MRI-DWI have been reported. These findings suggest that the clinicopathological spectrum of NIIDs should be elaborated upon (Tian et al., 2019; Miki et al., 2022). Additionally, in the present case, the gastrointestinal symptom of intestinal pseudo-obstruction was observed during the disease. Autonomic symptoms including urinary disturbance, miosis, orthostatic hypotension, arrhythmia, and gastrointestinal dysfunction are seen in NIID patients (Sone et al., 2016; Lu and Hong, 2021). To date, some NIID cases presenting with chronic idiopathic intestinal pseudo-obstruction have been reported (Sone et al., 2005; El-Rifai et al., 2006; Yamaguchi et al., 2018). Evidently, this is not entirely plausible since gastrointestinal hypomotility can also be seen in the elderly and long-term enteral feeders. However, based on the presence of autonomic symptoms, NIID may be considered.

In three previously reported cases, the coexistence of NIID and ALS was confirmed on autopsy in patients originally diagnosed with ALS, which are compared in an additional table (see Supplementary material 2) (Kakita et al., 1997; Seilhean et al., 2004; Sugiyama et al., 2021). All of these cases had ALS pathology, such as TDP-43 pathology or Bunina bodies, as well as neuronal intranuclear inclusions. As in the present case, all those cases were clinically of the spinal form, which begins with asymmetric weakness in a limb and gradual worsening of weakness to the contralateral limb or other spinal areas within a year. The reported time to tracheostomy and invasive ventilation (TIV) and death ranged from 5 to 22 months, and the overall course ranged from 18 to 22 months, similar to the median time to TIV and survival (25.9 and 25–36 months, respectively) found in the spinal form. In contrast, in our case, the time to TIV and time of overall course were 48 and 10 months, respectively, and the rate of progression was slower. The distribution and course of muscle weakness were consistent with the progression of classical ALS; notably, our case had marked lower motor neuron signs but unremarkable upper motor neuron symptoms.

This case study has a few limitations. We could not determine the GGC-repeat length in *NOTCH2NLC* or the CGG repeat length in *FMR1*, although Fragile X-associated tremor/ataxia syndrome has similar pathology to NIID. The negative *FMR1* staining and the fact that nuclear inclusions were not found in Purkinje cells but were observed in neurons and systemic organs supported the diagnosis of NIID. Another limitation was that skin biopsy was not performed. Previously, GGC-repeat expansion in *NOTCH2NLC* was reported in 4 (0.73%) of 545 patients clinically diagnosed with ALS, which led the authors to suggest that ALS may be a specific phenotype of NIID (Yuan et al., 2020). The presence of nuclear inclusions in skin biopsy was confirmed in 50% of these patients; however, these observations were not confirmed with postmortem histological analysis (Yuan et al., 2020). We suggest performing skin biopsy on patients who present with a classical ALS course but slow progression with predominant lower motor neuron symptoms.

## Conclusion

Here, we described a case of NIID with an ALS phenotype. We suggest that a subtype of sporadic NIID may exist that fulfills the diagnostic criteria for ALS.

## Data availability statement

The original contributions presented in the study are included in the article/Supplementary material, further inquiries can be directed to the corresponding author.

## Author contributions

MF, TU, AA, and MT collected and analyzed the data. MF, TU, YM, and KW drafted the manuscript, helped write, and revise the manuscript. YM and KW performed histological examination and designed the study. HK performed the pathological examination. All authors read and approved the final manuscript.

## Funding

This work was supported by JSPS KAKENHI [Grant Nos. 21K07452 (YM), 22H02948(KW)] and the Sakurai Memorial Fund for Medical Research (YM).

## Conflict of interest

The authors declare that the research was conducted in the absence of any commercial or financial relationships that could be construed as a potential conflict of interest.

## Publisher's note

All claims expressed in this article are solely those of the authors and do not necessarily represent those of their affiliated organizations, or those of the publisher, the editors and the reviewers. Any product that may be evaluated in this article, or claim that may be made by its manufacturer, is not guaranteed or endorsed by the publisher.

## Abbreviations

ALS: Amyotrophic lateral sclerosis
CNS: Central nervous system
DWI: Diffusion-weighted imaging
FUS: Fused in sarcoma
FTLD: Frontotemporal lobar degeneration
MRI: Magnetic resonance imaging
MUP: Motor unit potential
NIID: Neuronal intranuclear inclusion disease
NIID-D: Neuronal intranuclear inclusion disease-dementia
NIID-M: Neuronal intranuclear inclusion disease-limb weakness
NIID-P: Neuronal intranuclear inclusion disease-parkinsonism
*NOTCH2NLC*: Notch 2 N-terminal like C
TIV: Tracheostomy and invasive ventilation.
